# Why the role of mHealth in allergy diagnosis and treatment adherence cannot be overlooked

**DOI:** 10.1002/clt2.12298

**Published:** 2023-10-17

**Authors:** Anna Szylling, Filip Raciborski, Oksana Wojas, Konrad Furmańczyk, Edyta Krzych‐Fałta, Jean Bousquet, Boleslaw Samoliński

**Affiliations:** ^1^ Department of Allergy and Clinical Immunology University Clinical Center of the Medical University of Warsaw Central Clinical Hospital Warszawa Mazowieckie Poland; ^2^ Department of Prevention of Environmental Hazards, Allergology and Immunology Medical University of Warsaw Warszawa Mazowieckie Poland; ^3^ Institute of Information Technology Warsaw University of Life Sciences Warszawa Poland; ^4^ Department of Basic of Nursing Medical University of Warsaw Berlin Germany; ^5^ Institute of Allergology Charite Universitatsmedizin Berlin Berlin Germany; ^6^ University of Montpellier Montpellier France

**Keywords:** allergic rhinitis, allergy diagnosis, CDSS, mHealth, RWD, SWOT analysis

## Abstract

**Background:**

Allergic diseases—rhinitis and asthma—are the most common chronic conditions affecting adults. Traditional approaches to allergy diagnosis and treatment do not meet the health needs of all patients. Treatment adherence remains a challenge for physicians. The ubiquity of Internet access paired with limited in‐person contact with medical personnel in the course of the COVID‐19 pandemic demonstrated the potential of mHealth in communicating health information.

**Body:**

The abundance of new applications dedicated to various medical specialties encourages reflection on the informed use of such tools. The paper takes a closer look at the potential of mHealth and presents conclusions of selected studies focusing on the use of good apps. The strength weakness opportunities threats analysis was used to illustrate the strengths of the mHealth strategy, as well as its advantages, limitations and areas in need of further development.

**Conclusion:**

The strength of mHealth depends on the quality and quantity of the collected patient data, its reliable processing, as well as publication of outcomes and conclusions from analyses. Therefore, it is necessary to promote the use of validated applications among patients, physicians and medical staff.

## 1 INTRODUCTION

Allergic rhinitis (AR) is one of the most common chronic conditions in the world.^1^ It often coincides with asthma or conjunctivitis, affects patients' quality of life, disrupts social and professional functioning, as well as education, generates financial costs and burden to the healthcare system, worsens worker productivity and correlates with poorer educational performance of pupils and students.^2^, ^3^

The traditional diagnostic regimen based on medical history, spot skin tests and allergen‐specific IgE determination results in the implementation of therapies compliant with current clinical trial‐based recommendations, yet not always resulting in satisfactory improvement of particular patient's condition.^4^ With mobile technology, we are able to rapidly acquire and process up‐to‐date, real‐world‐data (RWD), shedding new light on the phenotyping of AR patients, on allergic comorbidity and treatment efficacy evaluation, allergen immunotherapy (AIT) in particular.

Availability of mobile phone networks in Poland far exceeds that of landline services. Per capita 148% of Poles own a mobile phone, while 12.4% have landlines, meaning that everyone owns at least one cell phone. Mobile network coverage in Poland is the highest among EU Member States, and significant capacity of the data transfer system further contributes to the broad use of mobile Internet.^5^ Given the rising cost of healthcare in chronic diseases and the shortage of medical personnel, accompanied by the rapid development of medical technology, a shift toward mHealth seems inevitable.^6^ In anticipation of reliable, certified apps, there is a risk that patients hoping to solve their medical problems will turn to available yet unregulated software.^7^

**FIGURE 1:**
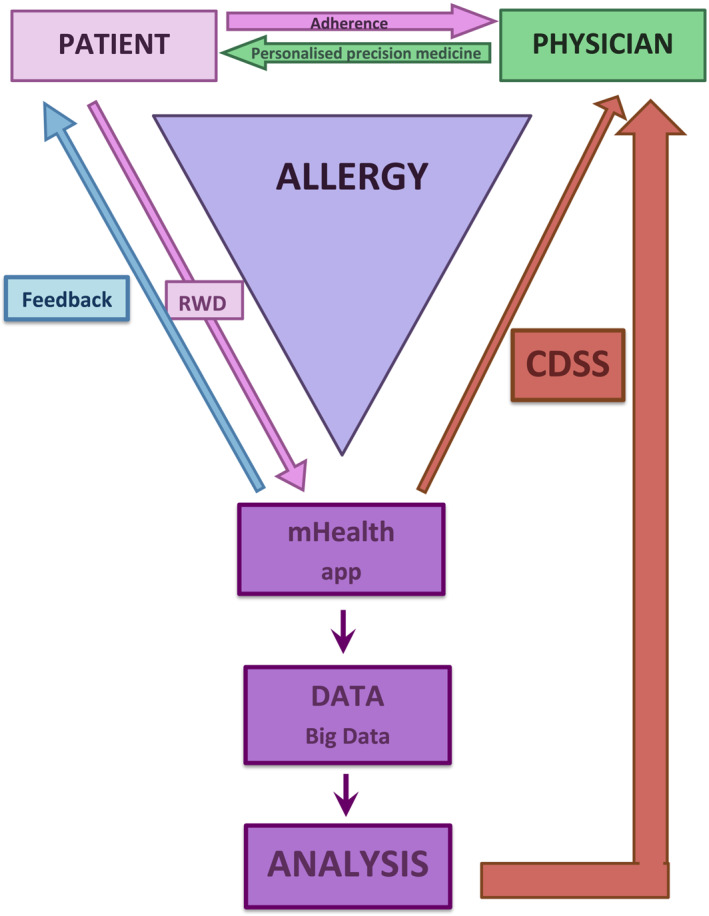
Information flow chart—mHealth supports the physician in personalized precision medicine.

Information flow chart—mHealth supports the physician in personalized precision medicine (Figure 1):

- When a PATIENT uses an app, new data (RWD) is generated regarding their health status, symptom severity, medication intake, general well‐being and work or school activity. Patients are able to self‐monitor allergy symptom control and modify therapy; they also receive incentives encouraging further use of an app, as well as congratulations after each completed activity (Feedback).
- DATA from multiple apps is recorded anonymously and creates Big Data; conclusions from the analyzed population data are global in scope.
- Clinical decision support systems (CDSS) is based on individual patient data from mHealth and/or Big Data analysis.
- PHYSICIAN utilizes mHealth, considers patient's individual reports and/or Big Data analysis, working toward personalized precision medicine, evaluates treatment adherence. At each medical visit, the patient is encouraged to use an app regularly, and thus to implement a tailor‐made treatment plan.

## 2 mHEALTH

mHealth refers to the use of mobile wireless devices for support of the treatment process.^8^, ^9^ It is a component of digital Health,^10^ eHealth (electronic health) utilizes information technology for healthcare, including telemedicine, apps, and Big Data analysis.^11^, ^12^, ^13^

After launching the app, the users provide their biometric information, for example, age, sex, date and time of use and current location. Despite their anonymity, the data is what makes the user unique. The next step is “playing a game” of answering questions, selecting certain patterns of responses. The user of a medical app becomes a source of data, which, following analysis, provides information to the Data center. Owing to Big Data analysis and processing, new epidemiological data is being created, specific to the users of a given app.

The quantity and variety of input information, as well as frequency of use of the app, determine the quality of conclusions, profiling of topics and disease phenotyping.^12^, ^14^

There are several subject areas to be distinguished in the field of mHealth: personal, social, home health monitoring, public education, medical records, communication, and professional consults.^9^

For physicians, the use of mHealth strategies in clinical practice provides reliable information on the implementation of the prescribed treatment, improves adherence, and provides opportunities to support patient education. From the patient's perspective, it reinforces adherence to treatment, contributes to better disease control, improves patient's and caregiver's quality of life, educates on symptoms, encourages health‐promoting behaviors and self‐management. To a researcher, mHealth improves adherence in clinical trials and offers insight into the reasons for non‐adherence.^12^, ^15^

Actively involving the patient in daily evaluation of their own symptoms and intake of medication is likely to have favorable impact on their health‐related needs being more accurately identified and met, and on better adherence,^16^ which still calls for tireless efforts on the part of medical community and leaves room for the use of new technologies.^17^, ^18^

Viera et al. confirmed the reliability of RWD collected with Mask‐air® app versus Google Trends, based on the report on antihistamines taken by patients in 10 EU countries. The author notes the possibility of the sample being unrepresentative (only app users were included), but stresses the importance of this data, which can be individually analyzed with consideration for disease severity.^19^

CDSS are software algorithms supporting healthcare providers in the process of diagnosis and patient management. They are based on an interaction between patient data and medical information (such as prescribed medication). CDSS should be founded on the best evidence and algorithms to assist patients and medical professionals in their joint efforts to shape treatment strategy providing for optimum control.^8^ CDSS consist of the following elements: data, algorithms and reporting.^20^ It should be noted that digital approaches to disease management must be founded on strong evidence, have a robust infrastructure, be designed collaboratively as clinically effective and cost‐effective, and reflect the needs of patients and providers.^21^

Professor Bousquet has been presenting evidence on the importance of patient communication technology for many years.^22^, ^23^ He has been pointing out the tremendous impact of information and communication technology on CDSS.^21^, ^24^ He demonstrates how the new type of data, RWD, is revising existing practice in the area of treatment and diagnosis, shaping new trends and contributing to better recommendations.^24^, ^25^, ^26^, ^27^, ^28^

What do we learn from Mask‐air® data analysis^29^

1. Mobile technology is becoming an important tool for better understanding and management of AR, providing novel input from daily patient follow up, RWD, never before available.^8^, ^22^, ^30^, ^31^, ^32^
2. The use of Visual Analogue Scale (VAS) to monitor daily AR symptoms proves to be a reliable tool in evaluating symptom severity and treatment efficacy.^33^
3. Sousa‐Pinto demonstrated a discrepancy when it comes to treatment adherence: for continued AR symptom control clinicians recommend long‐term treatment, while patients medicate on demand, when symptoms of AR develop or exacerbate. Multidrug therapy correlates with AR characterized by poorer AR symptom control.^22^
4. Similarly in Europe, patients tend to be non‐compliant and only take medication after symptoms develop. Multiple drug therapy is administered on one third of total treatment days, more frequently in the presence of severe AR symptoms, in particular during spring pollination season.^34^
5. Sousa‐Pinto demonstrated the usefulness and validity of Mask‐air® for the evaluation of AR treatment outcomes in terms of symptoms and medication use, as well as symptoms‐medication score. He recommends that Mask‐air® app should be employed to evaluate AIT efficacy, and as an element of clinical trials.^33^, ^35^
6. At present, there is no validated biological marker to anticipate AIT efficacy. Mobile health markers are likely to be helpful in confirming AIT success, not only under clinical trial conditions but in daily practice—for instance, in order to profile patient groups who need AIT and should be followed up for treatment efficacy.^28^
7. Sousa‐Pinto confirmed the efficacy of AIT sublingual immunotherapy (SLIT)‐tablet in patients allergic to grass pollen. He demonstrated that AIT (subcutaneus immunotherapy (SCIT) and SLIT) is effective in individuals allergic to grass pollen, the same effect was not, however, evidenced for SCIT alone. The author cautions that these reports require further investigation due to potential multifactorial relationships.^36^
8. In an Italian group, Ventura showed that elderly age was not a barrier to using Mask‐air®, and even individuals of low educational status were capable of operating the app after a brief training.^37^

## 3 NOTES ON THE mHEALTH TOOL

In 2018, Steurs analyzed the usefulness of mHealth in chronic respiratory conditions. Out of 314 apps, 112 were included in the analysis: 15 related to asthma and chronic obstractive pulmonary disease (COPD), 71 to asthma, 15 to COPD, 5 to asthma and AR and 6 to rhinosinusitis. The following topics were assessed:

1. Self‐monitoring: app user/patient provides information on symptoms and/or disease control, medication use, pulmonary function, use of inhaler; real‐time information on the environment and physical activity
2. Personalized feedback: daily review of reports with an option of presenting them to the physician, feedback, reminders to take medication and/or report symptoms.
3. Patient education.

With a maximum score of 10 points, mere 3% of apps scored ≥7, 28% scored 4–6 and 68% ≤3 points; only several apps were co‐created by physicians.^38^

In a systematic review on technology‐based interventions in asthma, Doshi analyzed 12 clinical trials focusing on better asthma control, patient education, monitoring of symptoms, controlling environmental factors responsible for exacerbations, treatment of comorbidities and adherence. The author stresses that standardized interventions are needed to better clarify the relationships between these factors.^39^

Chadran confirmed the educational value of mobile apps among healthcare professionals and students. App advantages include low cost, versatility and ability to use them anyplace, online or offline.^40^

In a meta‐analysis of systematic database reviews, Hui analyzed 12 randomized trials and 3 of them (25%) demonstrated an improved asthma control as assessed by ACQ (asthma control questionnaire). In the group exposed to intervention, there was a statistically significant improvement in asthma control (mean difference −0.25 [95% CI, −0.37 to −0.12]), although overall clinical efficacy of the apps, usually containing multiple functionalities, varied. Further research is needed to identify features related to adoption and adherence to mobile app use, as well as app characteristics that improve health outcomes.^41^

Cook revealed that integrated chronic disease management models using apps enable patient self‐care and facilitate shared treatment decisions, as well as improve adherence and asthma control: asthma control test (ACT) scores from 16.6 (inadequate to poor) to 20.5 (controlled) over the study period, 7.9% absolute increase in FEV1, reduction in the number of exacerbations requiring systemic corticosteroids over 6 months from 0.5 to 0.3, despite no need for daily follow up.^42^

Maintaining the attention of an app user is a difficult task for app developers and administrators, with the greatest number of confirmed entries on day one, when symptom severity is at its worst, as is AR control, and the patient decides to take medication. Mean time of use for Mask‐air® is 17.5 days, counting from the day of allergen exposure.^43^

An incentive system seems to have a favorable effect on adherence/adherence in children, with an improvement of 80% versus 33%, mean difference = 47%; 95% CI (33, 61), *p* < 0.001.^44^

## 4 RESULTS

Of the 29 families approached, 20 enrolled (69%). Participants were primarily Black (95%), publicly insured (75%) and averaged 2.9 asthma hospitalizations in the prior year. 15 of 16 caregivers (94%) surveyed at month 2 liked the idea of receiving adherence incentives. Mean adherence was significantly higher in month 1 compared with month 2 (80% vs. 33%, mean difference = 47%; 95% CI,^44^ *p* < 0.001).

In a paper by Kenyon, a group of 20 children aged 5–11, with high‐risk asthma, over the first month received a $1 incentive for daily complete adherence and ICS intake, while their parents obtained weekly feedback on their children's compliance. Caregivers reported that financial stimulus encouraged children to take inhaled medication. The study points toward the need for further work on the effectiveness of incentives encouraging adherence and administration of inhaled medication in high‐risk children.

In a literature review by Alqueran, interventional studies revealed a favorable impact of apps on asthma control among adolescents, as well as on adherence and own perception of treatment efficacy. Conclusions, however, are limited due to restricted sample size and lack of controls.^45^

Fedele, in a group of adolescents at high risk of loss of asthma control, found that the use of AIM2ACT app encouraged self‐management and improved adherence to therapy, while asthma control in the study sample improved in a statistically significant manner (*p* = 0.04).^46^, ^47^

Apps are readily used in trials with behavioral interventions, such as nicotine vaping cessation. For the intervention based on DynamiCare Health app, intervention components were rated favorably overall (>80%), yet statistical significance in the study group was not confirmed.^48^

In a study by Moor conducted in patients with uncontrolled asthma (ACT <20) (*n* = 437), who were randomized into 4 study arms and a control group, with follow‐up of 4–6 months, it was demonstrated that therapy supervision by means of sensors attached to inhalers (the connected inhaler system) and notifications sent to the doctor have the best effect on improving therapy adherence: 82.2 ± 16.5% (*n* = 83) as compared to controls 70.8 ± 27.3% (*n* = 85). No difference was evidenced between the treatment groups in terms of ACT. Mean percentage of rescue‐free days was at its highest if patients were aware of daily supervision over their primary and rescue medication intake and received doctor's feedback (monthly mean percentage of rescue‐free days, study arm 4 vs. 5 control, differences 7.3% [95% CI, 1.5%–13.2%], *p* = 0.015).^49^

Mosnaim demonstrated the impact of asthma self‐monitoring in patients (*n* = 100), who reported ICS and SABA administration via sensors integrated into inhalers, were supervised by a physician and received appropriate feedback. They maintained high adherence in terms of ICS use and cut down on the utilization of SABA (The percentage of SABA‐free days increased significantly in the treatment group [19%; 95% CI, 12–26; *p* < 0.01] and non‐significantly in the control group [6%, 95% CI, −3 to 16; *p* = 0.18], representing a 13% [95% CI, 1–26; *p* = 0 0.04] difference). ICS adherence changed minimally in the treatment group (−2%; 95% CI, −7 to 3; *p* = 0.40), but decreased significantly (−17%; 95% CI, −26 to −8; *p* < 0.01) in the control group, representing a 15% (95% CI, 4–25; *p* < 0 0.01) difference.^50^

In patients with uncontrolled asthma (*n* = 77, ACT<20), Ljungberg evidenced that additional data from the app, relating to correct inhalation technique or spirometry readings, tends to improve treatment outcomes, as compared to the group of patients with a paper‐based treatment plan (mean ACT difference 0.70, 95% CI, 0.06–1.34; *p* = 0.03).^51^ Improved medication adherence among the users of AsthmaTuner app was evidenced in the group of adult outpatients who used the app at least once a week (mean Medical Adherence Report Scale difference 0.45, 95% CI, 0.13–0.77; *p* = 0.01).

Nguyen's review of apps available in GooglePlay and Apple in the years 2007–2020 and dedicated to asthma control using sensors monitoring drug inhalation identified 2594 citations. After PubMed and Cochrane Central review, 7 studies involving 2 apps were included in the final analysis. Interventions were shown to slightly improve the use of maintenance medication inhaler and diminished the need for rescue inhaler, but failed to impact ACT scores. The author pointed to a need for a comprehensive evaluation of available apps and their impact on health outcomes before clinicians and patients can compare the benefits of adopting these technologies.^52^

The VitalFlow mobile spirometer measurements were highly correlated with nSpireKoKo® desktop device values in a group of adolescents with asthma (*n* = 48, 240 measurements), FEV_1,_ measurements (*r* ^2^ = 0.721, [95% CI, 0.749 ± 0.120], *p* < 0.001), and moderately correlated for FVC (*r* ^2^ = 0.617, [95% CI, 0.640 ± 0.130], *p* < 0.001). App‐based spirometer for home use is likely to improve asthma self‐monitoring.^53^

Composite interventions in a trial evaluating myAirCoach system in asthma patients (*n* = 30)—spirometry, nitric oxide levels, inhalation assessment, indoor air quality monitoring—favorably impacted asthma control (difference in ACQ was 0.70, *p* = 006), reduced exacerbations (6 incidents in the intervention arm vs. 12 in the control group (risk ratio 0.31; *p* = 0.06)); and elevated asthma‐related quality of life (minimum ACQ difference of 0.53; *p* = 0.04). However, emphasis is put on the need for validation of mHealth technology and further research in this field.^54^

The educational value of the ASTHMAXcel app (*n* = 30 users)—designed following user consultation, considering patient feedback, and in accordance with NAEPP, BTS/SIGN, and GINA guidelines—was compared against a control group (*n* = 30) subjected to training by an experienced educator, and confirmed effectiveness of the intervention in the Asthma Knowledge Questionnaire (AKQ). Mean AKQ in the ASTHMAXcel group versus human‐educator group pre‐intervention was 9.9 versus 10.5, *p* = 0.27, while mean AKQ post‐intervention in the ASTHMAXcel group versus human‐educator group was 12.3 versus 14.4, *p* = 0.0002. The ASTHMAXcel project failed to reveal differences in the training outcomes of the app versus educator.^55^

Hsia used the educational values of the ASTHMAXcel adventure, a gamified app, in a group of children with asthma (*n* = 39). The intervention took place during the initial patient visit and improvements were noted in asthma control at subsequent visits (from visit 1 to visit 2 and visit 3%–30.8% vs. 53.9%, *p* = 0.04; 30.8% vs. 59.0%, *p* = 0.02—affecting mostly boys), asthma knowledge and quality of life. There was also a reduction in ER visits and prednisone administration (from the initial visit to visits 2 and 3 [ED: 0.46 vs. 0.13, *p* = 0.03; 0.46 vs. 0.02, *p* = 0.02; prednisone use 0.49 vs. 0.13, *p* = 0.02; 0.49 vs. 0.03, *p* = 0.003]). In addition, a high patient satisfaction rate was recorded.^56^

In a randomized trial (*n* = 100), Sala‐Cunill demonstrated a behavioral change in patients at risk of anaphylaxis, following the use of an app and a smart case for an adrenaline auto‐injector. The author noticed an increase in satisfaction rate (VAS) after using the medical device versus prior to its use (89.1 [95% CI, 60.2–99.1] vs. 56.3 [95% CI, 48.1–81.4]; *p* < 0.0001), an improved adherence and a diminished sense of anxiety (from 52.2% to 29.3% [*p* < 0.001]), with 88% of patients additionally reporting having more influence on managing anaphylaxis.^57^

Baxter's meta‐analysis of studies on the use of intranasal corticosteroids in AR illustrates the problem the researchers are facing: diversity of apps and variable duration of interventions, which does not allow for unequivocal conclusions.^58^ This is why standardization, developing one uniform simple tool, becomes the most important thing in the world of mHealth. It is the only way toward thorough analysis and reliable, relevant conclusions.^58^

mHealth enables global medical consultations, fostering a virtual community where medical personnel share experiences freely. It plays a crucial role in disseminating essential information worldwide, enhancing access to medical care, and improving its quality.^59^

When evaluating any app, two main concerns are typically raised: protection of sensitive patient data and credibility of analyses collected via the app.

In 2012, the World Health Organization and the International Telecommunication Union launched a project entitled “Be He@lthy, Be Mobile(BHBM).” In collaboration with governments, the initiative was meant to foster targeted communication with service users on non‐communicable diseases and their risk factors. The new approach is to promote specific products, deliver a wide spectrum of health‐related and prevention‐related content worldwide, as well as integrate healthcare systems.^60^

Blakey points toward the need for standards governing digital interventions and strategies of dealing with large volumes of generated data.^61^ In allergology, positions by the American group (Elliot, ACAAI) pointed to the benefits of telemedicine for the doctor‐patient relationship and treatment adherence.^62^ The Europeans in turn (Matricardi, EAACI^63^) referred to the use of digital tools and their impact on the treatment process, mHealth. A 2016 analysis of 136 apps dedicated to AR, asthma, atopic dermatitis, chronic urticaria (CU), food allergy, anaphylaxis, allergies to medication or insect venom, noted their divergent content and quality, as well as limited number of clinically validated apps. It was also highlighted that many were not based on current recommendations. The evaluation took into account the benefits of use, utility values, efficacy and risks of employing them in allergic conditions. It was consequently pointed out that handling large volumes of generated data calls for certain regulation.^63^

Tripodi summarized 10 years of research on AllergyMonitor, an app for monitoring daily symptoms of seasonal AR, medication intake, AIT, as well as reporting adverse reactions to treatment with doctor's feedback specifying individual recommendations.

Studies with AllergyMonitor revealed the following:

1. The app is useful for diagnosing causes of AR in poly‐sensitized patients, by comparing the severity of recorded symptoms against pollen counts.
2. High adherence to app usage (>80%), as indicated and explained by the physician, is maintained for several weeks; confirmed role of the doctor in reinforcing adherence.
3. App use is likely to improve adherence to medication, including SLIT.
4. Selecting an optimum symptom‐severity‐score is important for a specific patient, not from the point of view of group evaluation.
5. In the short‐term evaluation of an individual patient, symptoms of seasonal AR could be anticipated on the basis of recorded symptoms and pollen counts.^64^

Dramburg confirmed a correlation between AllergyMonitor e‐diary use and AR severity on the population‐wide level. That effect is less clear in individual patient assessment.^65^

The ultimate goal in allergology is to precisely select causal treatment for allergy, namely allergen specific immunotherapy.^25^ Objective evaluation of the efficacy of the prescribed treatment, and its modification if need be, are the key tasks for a physician.^66^, ^67^ Patients with comorbidities and polysensitization constitute a challenge in the process of diagnosis and treatment.^68^, ^69^, ^70^, ^71^, ^72^ The doctor conveying benefits associated with reporting data via app and observing treatment recommendations is the essence of effective communication for better treatment adherence.^73^, ^74^

Features of an ideal app user, their demographic information including age, sex, educational status or occupation, remain a prime focus to researchers. Outlining the patient profile of an mHealth user can facilitate effective implementation of a treatment plan by the physician. On the other hand, the doctor's power of influence in their interaction with the patient, their familiarity with mHealth issues and ability to credibly recommend a particular app are likely to positively influence the use of this tool. Finally, reinforcing the message about app use at the next visit, reviewing previous outcomes and questioning the patient about his or her impressions of use so far, will undoubtedly influence treatment adherence.

We know the direction of app development for CU, and we know the needs of its users. Absence of a reliable CU app is well‐documented,^75^ and owing to the results of a cross‐sectional, multicenter UCARE study, we also know that half of the 1841 patients constituting the study sample were very much interested in monitoring their disease activity and disease control. One in 10, however, remains disinterested in the app.^76^ The study revealed that the majority of chronic utricaria patients utilize eHealth. They use communication systems, regularly review information on their disease, are interested in receiving news on chronic utricaria (average duration of disease in the investigated group is 3.7 years) and in communicating about it with the physician and fellow patients.^76^ Which is why a well‐designed app dedicated to this disease entity and its variants, taking into account co‐morbidities, assessing daily dynamics of lesions, QoL, triggering factors, impact on work and education, a tool validated on many platforms, would be likely to optimize the treatment of chronic utricaria in an individual patient. The data so compiled could prove useful for future research.^75^, ^76^

The cited studies show that mHealth apps can be successfully used in the treatment of allergies.

Strength weakness opportunities threats (SWOT) analysis is a valuable tool for mHealth research, informing decisions and optimizing impact in the dynamic digital health landscape.^77^ The SWOT analysis provided is quite comprehensive and highlights the key aspects of mHealth in allergy diagnosis and therapy adherence (Table 1).

**TABLE 1:** Features of mHealth strategy, SWOT analysis.^77^

	Positive	Negative
Internal factors App, application environment	1. Strengths RWD Big Data analysis, CDSS App updates Feedback: Alerts, reminders, gaming Symptoms control, self‐management Education Environmental influence on symptoms: Pollen, pollution	2. Weaknesses No localization, no app validation No analysis of collected data App without publication of results Lack/deficiency of environmental monitoring stations Information inconsistent with current standards in allergy treatment
External factors Patient	3. Opportunities Increasing number of users Number of days with the app Accuracy of user entries Promotion of the benefits of using the app	4. Threats Low variation in the user population Low user activity Data reliability Protection of privacy Data safety

Abbreviations: CDSS, clinical decision support systems; RWD, real word data; SWOT, strength weakness opportunities threats.

*Strengths*: mHealth offers significant potential with RWD and analytical capabilities, leading to clinically relevant conclusions and CDSS. It empowers patients with disease and treatment education, fostering self‐management and symptom control. It allows the assessment of environmental factors on well‐being and allergy symptoms.

*Weaknesses*: app validation and adaptation to local users are necessary. Analyzing collected data and publishing results is crucial. Limited environmental monitoring stations and substandard data may undermine the app's value.

*Opportunity*: increasing the number of app users willing to report on their health can enhance mHealth's impact. Physicians and patient advocacy groups can promote reliable apps, recruit new users, and encourage active participation.

*Threats*: limited user diversity and engagement, lack of diligence in data entries and availability of low‐quality medical app can pose changes. Ensuring transparent data protection policies is essential.

Overall, the SWOT analysis provides valuable insights into the potential of mHealth in allergy diagnosis and therapy adherence while identifying areas for improvement and growth.

## 5 CONCLUSIONS

The potential of the mHealth strategy in allergy diagnosis and treatment adherence, presented in the framework of a SWOT analysis (Table 1) (strengths, weaknesses, opportunities, and threats), reveals the complexity of features characterizing an ideal app. It is not easily tailored to the needs and capabilities of a patient.

The strength of mHealth depends on the quality and quantity of the collected patient data, its reliable processing, as well as publication of outcomes and conclusions from analyses.

Therefore, it is necessary to promote the use of validated applications among patients, physicians and medical staff.

## AUTHOR CONTRIBUTIONS

Anna Szylling wrote – original draft preparation, review and editing. Filip Raciborski, Oksana Wojas, Konrad Furmańczyk, Edyta Krzych‐Fałta participated in the redaction of the paper. Jean Bousquet is the chair of Mask‐air®, mHealth strategy mentor and educator, supervision. Boleslaw Samoliński participated in the design of the review and revised it critically, supervision. All the authors have reviewed and approved the manuscript.

## CONFLICT OF INTEREST STATEMENT

All authors have no conflicts of interest to declare.

## Data Availability

The data that support the findings of this study are available on request from the authors.
